# The Influences of Bioinformatics Tools and Reference Databases in Analyzing the Human Oral Microbial Community

**DOI:** 10.3390/genes11080878

**Published:** 2020-08-03

**Authors:** Maria A. Sierra, Qianhao Li, Smruti Pushalkar, Bidisha Paul, Tito A. Sandoval, Angela R. Kamer, Patricia Corby, Yuqi Guo, Ryan Richard Ruff, Alexander V. Alekseyenko, Xin Li, Deepak Saxena

**Affiliations:** 1 Department of Basic Science, New York University College of Dentistry, New York, NY 10010, USA; mas9996@nyu.edu (M.A.S.); kylin.qhl@outlook.com (Q.L.); sp117@nyu.edu (S.P.); bp1618@nyu.edu (B.P.); angela.kamer@nyu.edu (A.R.K.); patricia.corby@nyu.edu (P.C.); yg701@nyu.edu (Y.G.); xl15@nyu.edu (X.L.); 2 Department of Obstetrics and Gynecology, Weill Cornell Medicine, New York, NY 10065, USA; tas2037@med.cornell.edu; 3 Department of Epidemiology & Health Promotion, New York University College of Dentistry, New York, NY 10010, USA; ryan.ruff@nyu.edu; 4 The Biomedical Informatics Center, Program for Human Microbiome Research, Department of Public Health Sciences, Department of Oral Health Sciences, Department of Healthcare Leadership and Management, Medical University of South Carolina, Charleston, SC 29425, USA; alekseye@musc.edu; 5 S. Arthur Localio Laboratory, Departments of Surgery New York University School of Medicine, New York, NY 10016, USA

**Keywords:** 16S rRNA, databases, Greengenes, HOMD, NCBI, SILVA, RDP, QIIME, DADA2

## Abstract

There is currently no criterion to select appropriate bioinformatics tools and reference databases for analysis of 16S rRNA amplicon data in the human oral microbiome. Our study aims to determine the influence of multiple tools and reference databases on α-diversity measurements and β-diversity comparisons analyzing the human oral microbiome. We compared the results of taxonomical classification by Greengenes, the Human Oral Microbiome Database (HOMD), National Center for Biotechnology Information (NCBI) 16S, SILVA, and the Ribosomal Database Project (RDP) using Quantitative Insights Into Microbial Ecology (QIIME) and the Divisive Amplicon Denoising Algorithm (DADA2). There were 15 phyla present in all of the analyses, four phyla exclusive to certain databases, and different numbers of genera were identified in each database. Common genera found in the oral microbiome, such as *Veillonella*, *Rothia*, and *Prevotella*, are annotated by all databases; however, less common genera, such as *Bulleidia* and *Paludibacter*, are only annotated by large databases, such as Greengenes. Our results indicate that using different reference databases in 16S rRNA amplicon data analysis could lead to different taxonomic compositions, especially at genus level. There are a variety of databases available, but there are no defined criteria for data curation and validation of annotations, which can affect the accuracy and reproducibility of results, making it difficult to compare data across studies.

## 1. Introduction

With decreasing costs, speed improvements, and throughput of DNA-sequencing techniques, analyses using marker genes (e.g., 16S rRNA or 18S rRNA) have become one of the most common methods for studying microbial communities [1]. The human body hosts an abundant and complex diversity of microbial communities, denominated the microbiome [2]. The human microbiome has proven to be important in maintaining health, whereas dysbiosis is associated with various diseases and conditions [3]. Next-generation sequencing (NGS) technologies allow researchers to identify microbial taxa [4] and explore the possible role of the microbiomes in the human body [5].

Despite the wide use of 16S rRNA sequencing due to the latest advancements and benefits, errors and biases are introduced at different steps of the molecular experiment stage, from DNA extraction to sequencing, including amplification bias [6], chimeras [7], and biases introduced during computational analysis, such as Operational Taxonomic Unit (OTU) generation strategy, reference taxonomic sets, clustering algorithms, and specific software implementation [8,9]. Altogether, these methodologic differences could have dramatic effects on the accuracy of taxonomic classification, and α- and β-diversity estimation in 16S sequencing.

There are multiple bioinformatic tools available to analyze 16S rRNA gene amplicon sequencing data [10]. However, Quantitative Insights Into Microbial Ecology (QIIME) [11] and the Divisive Amplicon Denoising Algorithm (DADA2) [12] are among the most utilized [13]. Both pipelines are self-contained and can analyze 16S rRNA gene sequencing data from raw sequences (i.e., FASTQ), but they differ on how they cluster sequences: QIIME uses Operational Taxonomic Units (OTUs), sequences clustered with a fixed 3% dissimilarity threshold that might avoid fine-scale variation among sequences [14]. This method is used by most of the available pipelines [11,15,16,17,18]. Instead, DADA2 uses Amplicon Sequence Variants (ASV), an alternative error-modeling approach for denoising and clustering amplicons. Both pipelines enable the comparison of multiple and customized reference databases.

Taxonomic assignment is a crucial step in analyses. Reference databases are essential in the analysis of microbiomes because they are used to transform sequences into readable bacterial names. Reference databases for 16S taxonomy assignment include Greengenes [19], SILVA [20], the Ribosomal Database Project (RDP) [21], and the National Center for Biotechnology Information (NCBI) [22]). However, taxonomy assignment based on different reference databases might lead to different results [23].

The SILVA database contains information for all three domains of life (Bacteria, Archaea, and Eukarya). It is based on phylogenies for small subunit rRNAs (16S and 18S), and its taxonomic rank assignment is manually curated [24]. RDP database (Ribosomal Database Project) also contains rRNA sequences from the three domains, and most of the sequences are obtained from the International Nucleotide Sequence Database Collaboration (INSDC) [25]. Greengenes is a chimera-checked database that has Bacteria and Archaea sequences and most of the sequences are retrieved from the National Center for Biotechnology Information (NCBI) [26]. The NCBI taxonomy database contains the names of all organisms associated with submissions to the NCBI global database and is manually curated [22].

The human mouth harbors one of the most diverse and complex microbiomes in the human body, where up to 10,000 bacterial species have been identified [27]. Commensal taxa are associated with the development of dental caries and periodontal diseases [28], but it has also been associated with a higher risk of certain types of cancer (i.e., oral, pancreatic, gastrointestinal) [29,30,31]. Therefore, the standardization of bioinformatic tools and taxonomic reference databases for the analysis of the oral microbiome is key to correct taxa annotation to better understand the roles of oral microbiota in human health and disease. Here, we analyze the oral microbiome from 40 human saliva samples through two bioinformatic pipelines (i.e., QIIME and DADA2) and five reference databases (i.e., NCBI, Greengenes, SILVA, and RDP), including the Human Oral Microbiome Database (HOMD), which provides taxonomy for bacteria present in the human aerodigestive tract, including the oral cavity, pharynx, nasal passages, sinuses, and esophagus [32].

## 2. Materials and Methods

### 2.1. Collection of Samples

We collected saliva samples from 40 e-cigarette users. The Institutional Review Board of New York University Langone Medical Center approved the study, and all of the subjects provided informed consent and completed the questionnaires. Project identification code i16-00124: Impact of E-cigarette on Oral Health, approved on 3/2/2016. All subjects were initially screened for their carbon monoxide (CO) levels by an exhaled CO breath test (Smokerlyzer, Covita, Santa Barbara, CA, USA) and salivary cotinine levels using test strips (NicAlert, Craig Medical Inc., Vista, CA, USA) [33].

Periodontal health status was determined by a comprehensive oral examination and only subjects with mild to severe periodontal disease were included in the study. The inclusion criteria were as follows: aged at least 21 years; systemically healthy, as evidenced by medical history; and currently using e-cigarettes (never smoked and using 0.5 to 1 e-cig/day for past 6 months). They were diagnosed with mild, moderate, or severe periodontal disease, according to the CDC in collaboration with the American Academy of Periodontology (CDC-AAP) [34].

The exclusion criteria were: having a medical condition (including uncontrolled diabetes and HIV); subjects who reported taking antibiotics or having a professional dental cleaning within 1 month of the enrollment day; a recent febrile illness that delayed or precluded participation; pregnancy/lactation; enrollment in other studies; a history of radiation therapy to the head and neck region; the presence of oral mucosal lesions suspected of candidiasis; herpes labialis; aphthous stomatitis; and premalignancy/malignancy, such as leukoplakia or erythroplakias. The participants were asked to chew paraffin wax pellets (Gleegum, Verve Inc., Providence, RI) to stimulate salivary flow rate, and saliva samples were collected for 5 min. The saliva was aliquoted to a desired volume and stored at −80 °C until further analysis.

### 2.2. DNA Extraction and Sequencing

Genomic DNA was extracted from saliva samples using the MoBio Power fecal kit, following the manufacturer’s instructions (MoBio Laboratories Inc., Carlsbad, CA, USA). DNA was quantified for concentration and purity using a NanoDrop 2000 spectrophotometer (Thermo Scientific, Waltham, MA, USA) and stored at −20 °C until further analysis.

For high-throughput 16S library preparation and sequencing, the V3–V4 region of the 16S rRNA gene was amplified from the genomic DNA of saliva samples, according to the modified Illumina 16S metagenomics protocol (Part # 15,044,223 Rev. B). The purified DNA was quantified using the Quant-iT PicoGreen assay (Molecular Probes, Inc., Eugene, OR, USA) in a SpectraMax M5 microplate reader (Molecular Devices, Sunnyvale, CA, USA), and the concentrations were adjusted to 10 ng/µL for all sequencing assays. PCR was initially performed using the primer set, 341F (5′-CCTACGGGNGGCWGCAG-3′) and 805R (5’-GACTACHVGGGTATCTAATCC-3′), each with overhang adapter sequences (IDT, Coralville, IA, USA) using 2× Kapa HiFi Hotstart ReadyMix DNA polymerase (KapaBiosystems, Wilmington, MA, USA).

Samples were amplified in duplicates and purified using AMPure XP beads. Amplification was performed at 95 °C (3 min) with 25 cycles of 95 °C (30 s), 55 °C (30 s), 72 °C (30 s), and a final extension of 72 °C (5 min). Dual indices from Illumina Nextera XT index kits (Illumina, San Diego, CA) were added to target amplicons in a second PCR using 2× Kapa HiFi Hotstart ReadyMix DNA polymerase. PCR conditions were 95 °C (3 min), followed with 8 cycles of 95 °C (30 s), 55 °C (30 s), 72 °C (30 s), and a final extension of 72 °C (5 min). After each PCR cycle, AMPure XP bead-purified libraries were checked for purity using NanoDrop, quantified using PicoGreen assay, and size-confirmed on agarose gels. Negative controls were included in all sequencing runs. Equimolar amounts of the generated libraries were combined and quantified. The pooled amplicon library was denatured, diluted, and sequenced on an Illumina MiSeq platform using MiSeq Reagent Kit v3 (600 cycles), following the 2 × 300-bp paired-end sequencing protocol.

### 2.3. Bioinformatic and Statistical Analysis

QIIME scripts were run on the NYU High Performance Computing Cluster (HPC). Quality control of sequences was performed with FastQC [35].

QIIME v1.9.1 was used to process the data. Cutadapt (v1.12) [36] was used to remove the primers from both forward and reverse sequences. The cleaned sequences were merged using join_paired_ends.py, and barcodes were extracted from the sequence headers. The sequences were then pooled, de-multiplexed, and filtered for quality control using multiple_split_libraries_fastq.py. The open-reference OTU-picking method, pick_open_reference_otus.py, was used to create an OTU table with default settings. Sequences were aligned with parallel_align_seqs_pynast.py using the PyNAST default method. The Usearch61 method was used to perform de novo and reference-based chimera detection using the parallel_identify_chimeric_seqs.py, and the chimeric sequences were filtered out using filter_fasta.py. The alignment built with PyNAST was filtered to remove highly variable regions with filter_alignment.py, and the OTU table was filtered for chimeric OTUs with filter_otus_from_otu_table.py.

DADA2 v1.14 was used with the pipeline tutorial (available at https://github.com/benjjneb/dada2). Sequence reads were first filtered using DADA2’s default parameters (i.e., an expected error threshold of 2 with trimming of 250 and 200 bases for forward and reverse, respectively). Filtered reads were then de-replicated and de-noised using DADA2 default parameters. De-replication combines identical reads into unique sequences and constructs consensus quality profiles for each combined lot of sequences [37]. The consensus quality profiles then inform the de-noising algorithm, which infers error rates from samples and removes identified sequencing errors from the samples.

**Figure 1:**
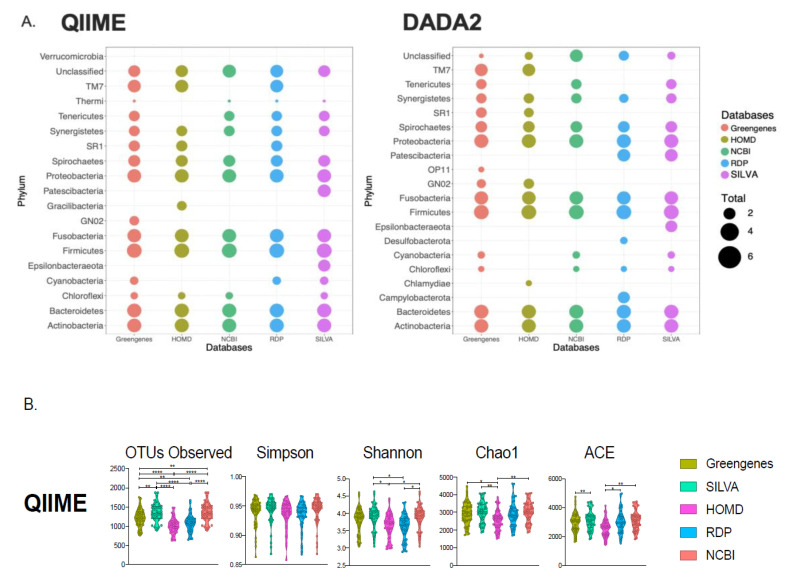
(**A**) Dotplot of phylum abundances from Quantitative Insights Into Microbial Ecology (QIIME) and the Divisive Amplicon Denoising Algorithm (DADA2) pipelines, comparing the five reference databases. Total abundances are log10 transformed. (**B**) α-diversity measurements for QIIME pipeline. *p*-values are assigned as ≤0.05 (*), <0.002 (**), <0.0002 (***), and <0.0001 (****).

The phyloseq (v1.27.0) [38] was used to format OTUs/ASVs tables and calculate diversities of samples. α-diversity measures, such as Richness (measured by Observed OTUs, Abundance-based Coverage Estimator (ACE), and Chao1) and Species evenness (measured by Shannon index), were calculated using phyloseq (v1.27) [38] and metagMisc (0.0.4) available is R. For α-diversity measures of QIIME output, no normalization was made. However, a log10 normalization was made on the total abundance at phylum level presented in Figure 1. Normality (Shapiro–Wilk Test) and one-way ANOVA were used to evaluate the significant differences in α-diversity measures in Prism 8 (GraphPad), with *p*-values ≤ 0.05 considered significant. For β-diversity comparisons, a prevalence heatmap of presence/absence at phylum level was built. Additionally, Non-metric Multidimensional Scaling (NMDS) was calculated at phylum and genus level using vegan (v2.5.4) [39] package in R (v3.6).

### 2.4. Reference Databases

The FASTA files and taxonomy tables of the 16S rRNA gene for each database were downloaded from their respective websites.

Greengenes (v13_8) was downloaded from each pipeline website.

QIIME: http://qiime.org/home_static/dataFiles.html

DADA2: https://benjjneb.github.io/dada2/training.html

HOMD (v15.1, updated at 11/16/2017) was downloaded from the eHOMD website (http://www.homd.org), starting from position 9.

The NCBI-curated collection of 16S rRNA RefSeq sequences from the bacteria and archaea Targeted Loci Project was downloaded on January 28, 2019. “33175[BioProject] OR 33317[BioProject]” was used to search in the NCBI Nucleotide database. All of the items (21,075 in total) were downloaded as a FASTA file. The taxonomic information was extracted and downloaded using the tool, entrez_qiime, available on GitHub (https://github.com/bakerccm/entrez_qiime).

SILVA (v132, released at 04/10/2018) 16S-only reference sequences and taxonomy were downloaded from the SILVA website for QIIME and DADA2.

The RDP (v11) aligned Bacteria FASTA file and taxonomy file were downloaded from the webpage and used for both pipelines, QIIME and DADA2 (https://rdp.cme.msu.edu/misc/resources.jsp).

### 2.5. Data Availability

The sequences generated and analyzed during the current study are available in the NCBI repository, accession number PRJNA602902.

## 3. Results

A total of 5,453,541 sequences from 40 samples were processed using QIIME and DADA2. QIIME took approximately 4.5 h to produce a raw OTU table, while it took DADA2 1.5 h to produce a raw ASV table. By using QIIME, 3,125,624 sequences remained after merging and demultiplexing. These sequences were clustered into Greengenes: 16,018, HOMD: 14,291, NCBI: 16,028, SILVA: 16,078, and RDP: 16,426 OTUs. By using DADA2 and each of the databases, 2,750,305 sequences remained after quality control, denoising and merging, which were clustered into 9264 ASVs.

### 3.1. Comparisons of Taxonomic Composition and Diversity from Different Databases

Different phyla were detected when assigning taxonomic classification using different reference databases in QIIME (Table S1) and DADA2 (Table S2). Although there were 15 phyla present in all the analyses, four phyla were only present in certain databases, which was the case for the phylum candidatus Patescibacteria, detected in the SILVA database from the QIIME pipeline (Figure 1A), and the phylum Chlamydiae, which was only present in the HOMD database in the DADA2 pipeline. Phyla Actinobacteria, Fusobacteria, Firmicutes, Proteobacteria, and Synergistetes were among the most abundant phyla and annotated in all five databases. There were, however, multiple unclassified taxa, especially from the QIIME pipeline, where all the databases retrieved an average of ~38,000 unclassified OTUs, with a minimum of 11,892, from Greengenes, and a maximum of 98,058, from the NCBI database. α-diversity measures also displayed these differences in databases when analyzing with QIIME pipeline (Figure 1B). The number of observed OTUs in all databases showed statistically significant differences. Shannon index, Chao1, and ACE also were different. Since the DADA2 clustering method is based on the sequence variance, it does not produce singletons; thus, standard α-diversity measures could not be calculated.

Non-metric multidimensional scaling (NMDS) depicts the differences of databases at phylum level in both pipelines (Supplementary Figure S1A,B). In QIIME, most databases cluster together, except for the SILVA database, which clusters apart. While using DADA2, taxonomic assignment with the RDP database is more dissimilar than with other databases.

### 3.2. Comparison of Taxonomic Annotation at Genus Level

**Figure 2:**
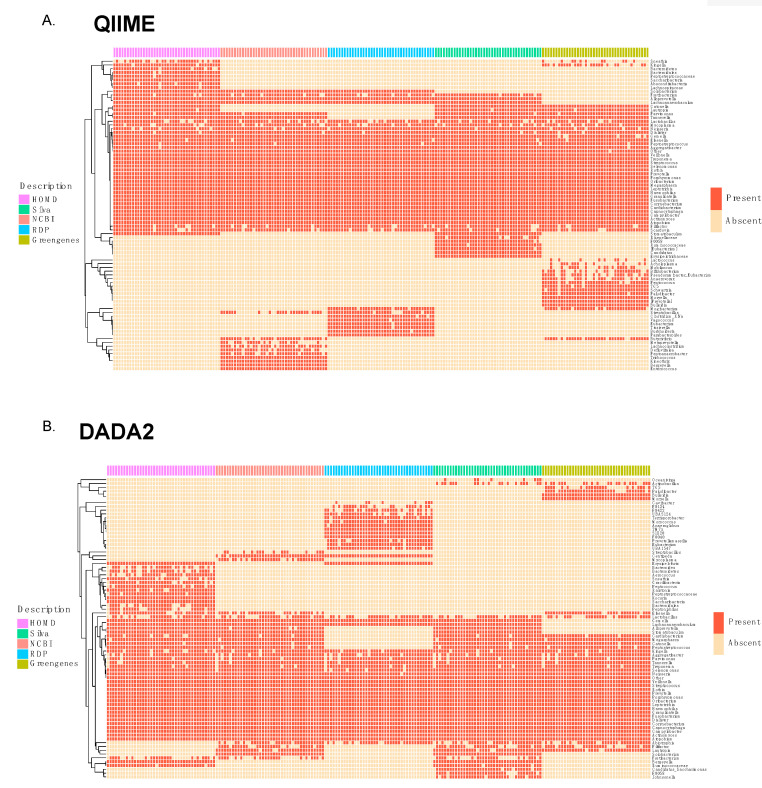
Prevalence heatmap of presence/absence of the 50 most abundant genera in (**A**) QIIME and (**B**) DADA2 pipelines.

Using the DADA2 pipeline, a total of 128 different genera were identified in the samples by Greengenes, 119 by HOMD, 127 by NCBI, 158 by SILVA, and 146 by RDP databases. As for the QIIME pipeline, 217 genera were retrieved with SILVA, 186 with RDP, 207 with NCBI, 119 with HOMD, and 175 with Greengenes. A prevalence heatmap displays the presence of approximately 30 genera in all databases in both pipelines (Figure 2A,B). These common taxa include genera such as *Veillonella*, *Rothia*, and *Prevotella*, among others. Some genera were only present in certain databases, such as *Bulleidia* and *Paludibacter*, which were only found when using the Greengenes database, and the genera, *Ralstonia* and *Aerococcus*, which were only annotated when using the HOMD database and the DADA2 pipeline. Depending on the database, some genera are named with numbers, which corresponds to isolated strains. This is the case for *TG5*, present in Greengenes and assigned to phylum Synergistetes, and *F0058*, present in SILVA and assigned to Bacteroidetes. These taxa have been related to oral microbiome studies and associated with dental disease [40,41].

**Figure 3:**
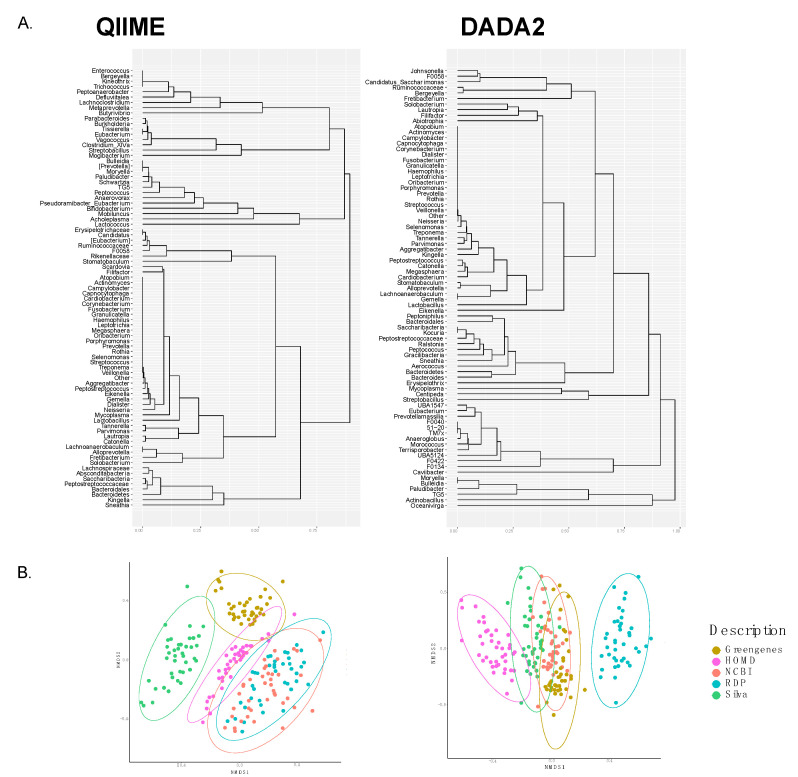
Comparisons of hierarchical clusters at genus level. (**A**) Cladogram of the 50 most abundant genera in each pipeline, with cophenetic correlation coefficients of *r* = 0.889 and *r* = 0.898 for QIIME and DADA2, respectively. (**B**) Non-metric multidimensional scaling (NMDS) at genus level, based on a Bray–Curtis dissimilarity matrix.

For each of the 50 most abundant genera, we built a tree for each of the two pipelines and calculated the cophenetic distance matrix (Figure 3A), measuring the distances between leaves of the phylogenetic tree through branch lengths, as implemented in the R package stats. Values of correlation were *r* = 0.889 and *r* = 0.898 for QIIME and DADA2, respectively. These differences were also evident when performing non-metric multidimensional scaling (NMDS) (Figure 3B). Using the QIIME pipeline, we found a greater dissimilarity of sample annotation, and even more when choosing the SILVA or Greengenes databases, while there was an evident clustering of samples annotated with NCBI and RDP. On the contrary, samples analyzed with DADA2 seemed not to be as affected by the choice of Greengenes, NCBI, or SILVA databases; however, RDP clustered apart, showing a greater dissimilarity.

## 4. Discussion

Over the last few decades, next-generation sequencing (NGS) has greatly improved investigations into complex microbial communities. The development of computational methods has also played an essential role in this process by enabling researchers to analyze and transfer sequencing data into human-readable results. In this study, we analyzed 16S data from human saliva samples using five different reference databases and two different bioinformatics pipelines to compare their influence in exploring the composition of oral microbiomes. Fifteen phyla were commonly detected by the five databases, although Actinobacteria, Fusobacteria, Firmicutes, Proteobacteria, and Synergistetes were among the most abundant and prevalent in all databases. The dissimilarities shown in the NMDS in both pipelines using certain databases at phylum level might be due to, for QIIME, the SILVA database not including phyla TM7 and SR1, which seem highly abundant in these oral samples when using other reference databases. Additionally, SILVA includes the candidatus Patescibacteria and the phylum Epsilonbactereaota, which has been classified as an independent phylum in some studies, but as a Proteobacteria class in others [42]. For DADA, the dissimilarity in the RDP database might be because it classifies Firmicutes in three additional groups, Firmicutes_A, Firmicutes_B, and Firmicutes_C, and does not include phyla TM7, GN02, or SR1, which are frequently detected in the human microbiome, including the human oral cavity [43].

Researchers should be cautious when choosing a particular database. Public databases might annotate multiple unverified phyla, as was the case with Campylobacterota and Desulfobacterota in RDP and Epsilonbactereota in SILVA, as well as create misannotations that could lead to false positives [44], which we suggest was the case with Verrucomicrobia, which was only annotated in two OTUs using Greengenes. Using custom databases and reducing the size of the reference database to the specific microbiome, one might avoid misannotations and improve taxonomic assignation at lower taxonomic levels [45]. However, a small reference database could skip certain taxa that have not yet been annotated, due to a lack of sufficient sequencing effort or a lack of previous isolation. As an example, the phylum Tenericutes was not annotated when using HOMD, but seemed highly present when using other databases.

When we compare the taxonomic annotations at genus level, half of the taxa were annotated independently of the database or the pipeline. These common taxa, which we denominated as “core-genera”, seem to be commonly associated with human commensals, especially from the oral microbiome [46]. Nonetheless, some genera are excluded from HOMD and other customized databases, as is the case for *Stomatobaculum* [47], *Lactococcus* [48], *Bulleida* [49], and *Vagococcus* [50]. Other genera, such as *Acholeplasma*, *Mobiluncus*, *Anaerovorax*, and *Prevotellamassilia*, that have not been broadly associated with the oral microbiome, were also omitted in HOMD and only annotated in public reference databases, which could suggest that this customized database should be further nourished with new taxa annotations.

The differences found in the genera assignation were shown in the cladogram, cophenetic distance, and NMDS. This depicts the influence that a reference database might have on taxonomic assignment at genus level. These differences were not as notable when working with a higher phylogenetic level (i.e., phylum), although, when analyzing the role of the microbiome in human health and diseases, it is crucial to identify microbial taxa as precisely as possible (i.e., genus or species level) [51].

Extracting valuable information from enormous amounts of 16S sequencing data requires not only high-quality reference sequences, but also accurate and validated annotations. As researchers continue to extensively use these open-source reference databases, improved quality control will be necessary during their curation and validation. NCBI might arise as a good option as a scaffold, due to its large-scale sources and daily updates. However, custom and up-to-date databases are encouraged in order to validate the veracity of taxonomic annotations, avoid misclassifications, and improve taxonomic assignation at lower taxonomic levels. Additionally, databases with validated taxonomy and phenotypic and ecological characterization of species found in the microbiome are essential to understanding the role of the microbes in the microbiome [52,53].

In our study, we also compared QIIME and DADA2 pipelines to analyze our dataset. In accordance with other studies [54], DADA2 identified fewer ASVs than the number of OTUs identified by QIIME. QIIME is used in most microbiome studies [55,56], and provides a Biological Observation Matrix (BIOM file), which is useful for a wide range of downstream analyses. However, DADA2 might be faster and easier for less-experienced users, and arises as an alternative error-modeling approach for denoising and clustering amplicons. Altogether, these data show that the reference database used to align sequences and assign taxonomy information can have an effect on the final results.

## 5. Conclusions

Our study showed that using different reference databases in 16S sequencing data analyses could lead to different taxonomic compositions, especially at genus level. Currently, there are a variety of databases available, but there are no defined criteria for data curation and validation of annotations. This could affect the accuracy and reproducibility of results, and also makes it difficult to compare data from other studies. A well-curated and up-to-date microbiome-specific database is needed to improve the reliability of 16S sequencing analyses and taxonomic annotations. The HOMD database might be a good start, but our results suggest that more taxa should be included.

We also compared QIIME and DADA2 in our study and found that at phylum level the choice of pipeline might not affect the results as much as at a lower level (i.e., genus), which could have a bigger impact on the results. Nevertheless, most of the common oral genera were present in both pipelines. Even though the number of OTUs (QIIME) was bigger than the number of ASVs (DADA2), our results show that the choice of certain databases might significantly affect the output, rather than the choice of pipeline. It is critical for researchers to consider these differences with regard to the questions they seek to answer and the type of microbiome under study.

## Supplementary Materials

The following are available online at https://www.mdpi.com/2073-4425/11/8/878/s1. Supplemental Figure S1: NMDS at phylum level of both pipelines based on a Bray–Curtis dissimilarity matrix. Supplemental Table S1: Phylum total abundance for each of the five databases in the QIIME pipeline. Supplemental Table S2: Phylum total abundance for each of the five databases in the DADA2 pipeline.
