# Supplementary material for: The Influences of Bioinformatics Tools and Reference Databases in Analyzing the Human Oral Microbial Community
Source: Genes (Basel). 2020 Aug 3;11(8):878. doi: 10.3390/genes11080878 (PMC7465726; doi:10.3390/genes11080878)
Supplement: Supplementary file 1 [file genes-11-00878-s001.zip › Supplemental/Figure S1.NMDS at phylum level of both pipelines.pdf]

Figure S1

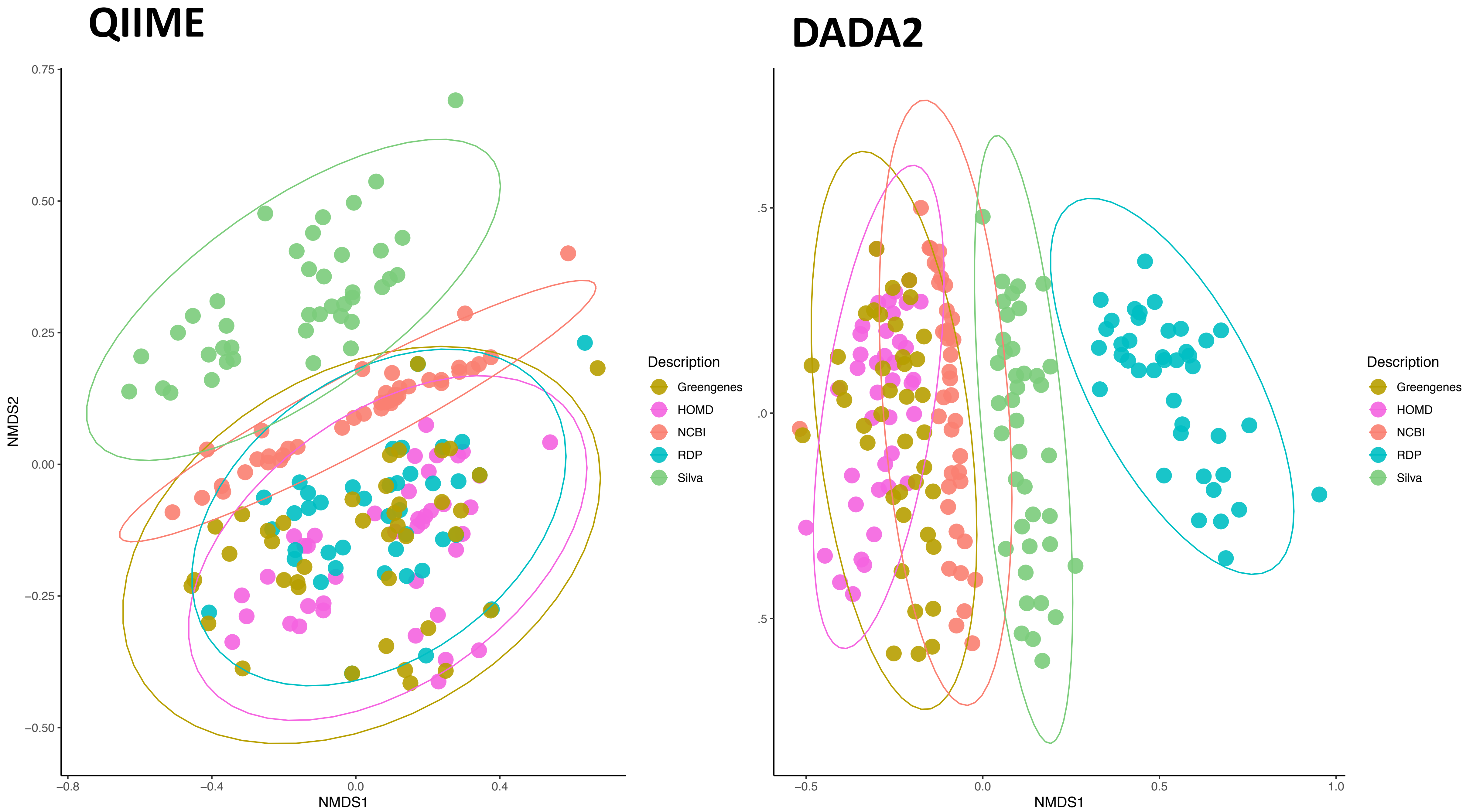
QIIME
DADA2
0.75
0.50
0.25
0.00
−0.25
−0.50
NMDS2
−0.8
−0.4
0.0
0.4
NMDS1
Description
Greengenes
HOMD
NCBI
RDP
Silva
.5
.0
.5
−0.5
0.0
0.5
1.0
NMDS1
Description
Greengenes
HOMD
NCBI
RDP
Silva

Table S1

Phylum	Total	Databases
Actinobacter	343561	SILVA
Actinobacter	346239	HOMD
Actinobacter	345438	Greengenes
Actinobacter	346543	NCBI
Actinobacter	346939	RDP
Bacteroidete	423354	SILVA
Bacteroidete	428181	HOMD
Bacteroidete	423927	Greengenes
Bacteroidete	416751	NCBI
Bacteroidete	408997	RDP
Chloroflexi	48	SILVA
Chloroflexi	48	HOMD
Chloroflexi	48	Greengenes
Chloroflexi	48	NCBI
Chloroflexi	0	RDP
Cyanobacteri	139	SILVA
Cyanobacteri	0	HOMD
Cyanobacteri	139	Greengenes
Cyanobacteri	0	NCBI
Cyanobacteri	138	RDP
Epsilonbacte	19960	SILVA
Epsilonbacte	0	HOMD
Epsilonbacte	0	Greengenes
Epsilonbacte	0	NCBI
Epsilonbacte	0	RDP
Firmicutes	1199367	SILVA
Firmicutes	1193193	HOMD
Firmicutes	1199462	Greengenes
Firmicutes	1171784	NCBI
Firmicutes	1195749	RDP
Fusobacteria	212411	SILVA
Fusobacteria	213027	HOMD
Fusobacteria	212283	Greengenes
Fusobacteria	211145	NCBI
Fusobacteria	216476	RDP
GN02	0	SILVA
GN02	0	HOMD
GN02	591	Greengenes
GN02	0	NCBI
GN02	0	RDP
Gracilibacter	0	SILVA
Gracilibacter	591	HOMD

Gracilibacter	0	Greengenes
Gracilibacter	0	NCBI
Gracilibacter	0	RDP
Patescibacte	63754	SILVA
Patescibacte	0	HOMD
Patescibacte	0	Greengenes
Patescibacte	0	NCBI
Patescibacte	0	RDP
Proteobacter	199092	SILVA
Proteobacter	217129	HOMD
Proteobacter	215837	Greengenes
Proteobacter	218972	NCBI
Proteobacter	217469	RDP
Spirochaetes	11927	SILVA
Spirochaetes	11843	HOMD
Spirochaetes	11919	Greengenes
Spirochaetes	11381	NCBI
Spirochaetes	10917	RDP
SR1	0	SILVA
SR1	3732	HOMD
SR1	3733	Greengenes
SR1	0	NCBI
SR1	3722	RDP
Synergistete:	2291	SILVA
Synergistete:	2278	HOMD
Synergistete:	2287	Greengenes
Synergistete:	2263	NCBI
Synergistete:	2276	RDP
Tenericutes	2327	SILVA
Tenericutes	0	HOMD
Tenericutes	2328	Greengenes
Tenericutes	1330	NCBI
Tenericutes	1330	RDP
Thermi	3	SILVA
Thermi	0	HOMD
Thermi	3	Greengenes
Thermi	3	NCBI
Thermi	3	RDP
TM7	0	SILVA
TM7	59527	HOMD
TM7	59461	Greengenes
TM7	0	NCBI
TM7	57664	RDP

Unclassified	13499	SILVA
Unclassified	19498	HOMD
Unclassified	11892	Greengenes
Unclassified	98058	NCBI
Unclassified	50561	RDP
Verrucomicro	0	SILVA
Verrucomicro	0	HOMD
Verrucomicro	2	Greengenes
Verrucomicro	0	NCBI
Verrucomicro	0	RDP

Table S2

Phylum	Total	Databases
Actinobacter	350640	SILVA
Actinobacter	350668	HOMD
Actinobacter	350639	Greengenes
Actinobacter	350634	NCBI
Actinobacter	350634	RDP
Bacteroidete	506401	SILVA
Bacteroidete	506424	HOMD
Bacteroidete	506401	Greengenes
Bacteroidete	506417	NCBI
Bacteroidete	506413	RDP
Campylobact	0	SILVA
Campylobact	0	HOMD
Campylobact	0	Greengenes
Campylobact	0	NCBI
Campylobact	17128	RDP
Chlamydiae	0	SILVA
Chlamydiae	31	HOMD
Chlamydiae	0	Greengenes
Chlamydiae	0	NCBI
Chlamydiae	0	RDP
Chloroflexi	31	SILVA
Chloroflexi	0	HOMD
Chloroflexi	31	Greengenes
Chloroflexi	31	NCBI
Chloroflexi	31	RDP
Cyanobacteri	86	SILVA
Cyanobacteri	0	HOMD
Cyanobacteri	86	Greengenes
Cyanobacteri	86	NCBI
Cyanobacteri	4	RDP
Desulfobacte	0	SILVA
Desulfobacte	0	HOMD
Desulfobacte	0	Greengenes
Desulfobacte	0	NCBI
Desulfobacte	82	RDP
Epsilonbacte	17128	SILVA
Epsilonbacte	0	HOMD
Epsilonbacte	0	Greengenes
Epsilonbacte	0	NCBI
Epsilonbacte	0	RDP
Firmicutes	1373936	SILVA
Firmicutes	1376060	HOMD
Firmicutes	1373938	Greengenes
Firmicutes	1373927	NCBI

Firmicutes	1376075	RDP
Fusobacteria	190139	SILVA
Fusobacteria	190139	HOMD
Fusobacteria	190133	Greengenes
Fusobacteria	190140	NCBI
Fusobacteria	190138	RDP
GN02	0	SILVA
GN02	2114	HOMD
GN02	494	Greengenes
GN02	0	NCBI
GN02	0	RDP
OP11	0	SILVA
OP11	0	HOMD
OP11	27	Greengenes
OP11	0	NCBI
OP11	0	RDP
Patescibacte	57303	SILVA
Patescibacte	0	HOMD
Patescibacte	0	Greengenes
Patescibacte	0	NCBI
Patescibacte	57325	RDP
Proteobacter	386137	SILVA
Proteobacter	405843	HOMD
Proteobacter	403407	Greengenes
Proteobacter	404153	NCBI
Proteobacter	386129	RDP
Spirochaetes	10482	SILVA
Spirochaetes	10466	HOMD
Spirochaetes	10484	Greengenes
Spirochaetes	10484	NCBI
Spirochaetes	10484	RDP
SR1	0	SILVA
SR1	875	HOMD
SR1	3236	Greengenes
SR1	0	NCBI
SR1	0	RDP
Synergistete:	1910	SILVA
Synergistete:	1910	HOMD
Synergistete:	1910	Greengenes
Synergistete:	1910	NCBI
Synergistete:	556	RDP
Tenericutes	2137	SILVA
Tenericutes	0	HOMD
Tenericutes	2137	Greengenes
Tenericutes	2116	NCBI

Tenericutes	0	RDP
TM7	0	SILVA
TM7	51779	HOMD
TM7	53546	Greengenes
TM7	0	NCBI
TM7	0	RDP
Unclassified	151	SILVA
Unclassified	172	HOMD
Unclassified	12	Greengenes
Unclassified	56583	NCBI
Unclassified	1482	RDP
